# Informing children citizens efficiently to better engage them in the fight against COVID-19 pandemic

**DOI:** 10.1371/journal.pntd.0008828

**Published:** 2020-11-04

**Authors:** Jean-Eric Ghia, Sophie Gaulin, Laure Ghia, Laure Garancher, Claude Flamand

**Affiliations:** 1 Departments of Immunology and Internal Medicine, Section of Gastroenterology, IBD Clinical and Research Centre, Children’s Hospital Research Institute of Manitoba, Rady Faculty of Health Sciences, University of Manitoba, Winnipeg, Manitoba, Canada; 2 La Liberté Journal, Winnipeg, Manitoba, Canada; 3 Winnipeg School Division, Winnipeg, Manitoba, Canada; 4 The Ink link Association, Paris, France; 5 Epidemiology Unit, Institut Pasteur in French Guiana, Cayenne, French Guiana; Medizinische Universitat Wien, AUSTRIA

## Abstract

Since the beginning of the year, the world’s attention has rightly been focused on the spread of the Coronavirus Disease 2019 (COVID-19) pandemic and the implementation of drastic mitigation strategies to limit disease transmission. However, public health information campaigns tailored to children are very rare. Now more than ever, at a time when some governments are taking populations out of lockdown and youth are returning to schools, children around the world need to fully grasp the modes of transmission of the disease, the health risks, the scientific notions of the immune system, the value of barrier measures, and the progress of scientific research. In the context of the COVID-19 pandemic, comics can be very useful for communicating quickly and effectively abstract and important information to children who might be under the influence of a large amount of sometimes contradictory information. Conveying precise, reliable, and accessible information to children is key in a world overwhelmingly impacted by the outbreak. This should be the role and the responsibility of world health official leaders and governments in compliance with the United Nations Convention on the Rights of the Child. In partnership with mainstream medias, consortia of scientists, communication experts, and education specialists, it is urgent that world leaders engage children in this worldwide public health fight.

Since the beginning of the year, the world’s attention has rightly been focused on the spread of the Coronavirus Disease 2019 (COVID-19) pandemic and the implementation of drastic mitigation strategies to limit disease transmission [1,2]. The health, social, and economic impact of the outbreak combined with the unprecedented scale of media coverage focusing on the epidemic dynamics represents a very first in the history of public health. In this context, children are facing an overload of information about the COVID-19 outbreak, combining individual concerns, social conversations, mainstream media, and social media. The quality and reliability of this information is uneven. Sometimes, children witness conversations filled with irony and cynicism or can be faced with age-inappropriate scientific notions and vocabulary. While the magnitude of the health crisis related to COVID-19 has extended, some of the debates once reserved for scientists and associated with their specialized jargon have become accessible to a large part of the general population. Still, public health information campaigns tailored to children are very rare.

When governments choose mandatory and/or complete lockdown in the context of a health emergency, they leave it up to the parents to explain the whys and wherefores of their official decisions. Even though some interesting initiatives have emerged relatively soon after the beginning of the pandemic [3–5], very little or none at all direct, accessible, and frequent governmental communication was geared toward them. With physical lockdown, children are faced with information filtered through their parents and have little access to human and material resources to challenge them, like in a school environment. In this context, it is fair to say that children are also experiencing a kind of intellectual lockdown on reliable information. The quantity of information received does not always meet the standards of quality and reliability that children need in order to become safely empowered.

It is particularly important that clear, accessible, guilt-free, and reliable key information be transmitted to children to enable them to better understand their role in an ever-changing environment, engage them fully in the fight against the COVID-19 pandemic, and empower them with knowledge to prevent unnecessary anxiety, guilt, or even an unsafe careless attitude toward danger [6].

In this fast-evolving pandemic crisis, governments around the world should consider children as full-right citizens who need to be addressed daily, officially, and directly. According to the United Nations Convention on the Rights of the Child, access to information is an essential right: “States Parties recognize the important function performed by the mass media and shall ensure that the child has access to information and material from a diversity of national and international sources, especially those aimed at the promotion of his or her social, spiritual and moral well-being and physical and mental health” [7].

Communicating with children about such a sensitive topic requires a high level of pedagogy and accessibility.

Despite some initial resistance [8,9], the potential of comics as an educational tool has rapidly been recognized in the fields of education and psychology [10,11]. In recent decades, comics have indeed been used for public education purposes, and their use has been seen as a medium of choice as not only are they popular, but they can, when properly adapted, also act as a link to help children understand complex, nonfictional, and difficult concept or theme [10–15]. More recently, several empirical studies in the field of graphic medicine [16–18], on the use of comics, have shown that comics contribute to an overall improvement in community engagement on healthcare and medical issues [11,19–21]. Illustrations can represent objects and situations that are usually non-visible but also abstract concepts through visible metaphor. That will allow the reader to acquire new concepts to understand complex scientific and medical notions, but to be impactful, it is critical to question first the abstraction ability of the audience involving it in the design process [22]. For example, expecting a young child to understand that a “fun” image of a virus represents in fact a microscopic non-visible element is not obvious. A good comic relies on both a solid script combined with attractive and simple-to-read images. Both need to be tested on the audience to ensure that the message is properly transmitted. To do so, a participatory approach is key. That requires first writing a script while keeping in mind the abstraction capacity of the age group (for example, logigram, use of arrows, and changes in scale are not obvious) and then preparing initial sketches and asking children of different ages to explain what they see and what they understand and making corrections to ensure that the right message has been conveyed. When done properly, those comics can convey strong and complex messages in a nonthreatening and accessible way. Well-designed comics also have to rely on the use of characters and situation models, which provide the basis for emotional attachment and self-reference, which can also facilitate the formation of new memories [11] and potentially transcend language and literacy barriers.

In the context of the COVID-19 pandemic, comics can be very useful for communicating quickly and effectively abstract and important information to children who might be under the influence of a large amount of sometimes contradictory information.

The use of appropriate governmental communication using comic books, cartoons, appropriate websites, and social media would then make it possible to raise awareness among children about the modes of transmission of the disease, the health risks, the scientific notions of the immune system, the value of barrier measures, and the progress of scientific research. More than texts, comics could be better suited for information campaigns on social media like TikTok [23] and other popular apps. Ideally, they would be tailored by governments, scientific consortium, and educational experts to reach younger citizens.

While some governments are taking populations out of lockdown and children are returning to schools, it is urgent to set up consortia of scientists, public health experts, and communication, literacy, and education specialists and even engage social media companies to work together to meet the needs of children as full-class citizens, whose mental and physical health is as equally important as adults.
